# Erratum: Overweight and obesity among children and adolescents in Germany. Results of the cross-sectional KiGGS Wave 2 study and trends

**DOI:** 10.25646/6797

**Published:** 2018-07-19

**Authors:** A Schienkiewitz, AK Brettschneider, S Damerow, A Schaffrath Rosario

Schienkiewitz A, Brettschneider AK, Damerow S, Schaffrath Rosario A (2018)

Journal of Health Monitoring 3(1): 15-22.

DOI 10.17886/RKI-GBE-2018-022

In the original article, the number of cases of the KiGGS baseline study and KiGGS Wave 2 were switched in the titles of Figure 1 and Figure 2 on page 20. The number of cases was corrected in both figure titles: KiGGS baseline study n=7,215 girls, n=7,531 boys; KiGGS Wave 2 n=1,799 girls, n=1,762 boys.

The corrected version of the article is available at DOI 10.17886/RKI-GBE-2018-022.2

